# 

**Published:** 1889-01-12

**Authors:** 


**Notice to Advertisers and Subscribers.**

All Advertisements, Orders for Copies of THE HOSPITAL, and Business Communications should be addressed to The Publisher, *not to the Editor*, THE HOSPITAL, 38, Old Jewry, E.C.

**To Residents, Secretaries, and Matrons.**

The Editor will be glad to have the Regulations and each Report as issued by Asylums, Hospitals, Convalescent and Nursing Institutions, as well as those of other Charities, and an early copy in duplicate of all Appeals and Festival Notices, etc., addressed to *The Editor, The Lodge, Porchester Square, London, W.* Any superintendent, secretary, matron, or other chief official who regularly sends such information may be registered, on application to the Publisher, to receive free of cost a cover for binding THE HOSPITAL in half-yearly volumes.

A Scale of Charges will be found on the first page of the NURSING SUPPLEMENT.

### SCRAPS AND GLEANINGS.

MADRID is now suffering severely from an epidemic of diphtheria.

THE number of patients treated last year at the Calcutta Hospital was 263,849.

THE total amount of income tax refunded to charities during 1886-87 was £2,051,962.

THE Milan Hospital is probably the largest in the world: it receives over 3,000 patients.

THE patients treated at the Manchester Royal Eye Hospital during last year numbered 16,748.

THE Queen has sent presents of cast linen to the Grosvenor Hospital and other metropolitan institutions.

DR. B. W. RICHARDSON received the guests of the Cyclists' Society at the Conduit Street Galleries last week.

THE Mayor of Bradford gave a Christmas dinner to the patients, staff, nurses, and servants of the Infirmary.

THE Edinburgh Provident Dispensary is now lighted by electricity supplied from the chemical laboratory.

SIXTY-FIVE cases of scarlet fever were treated in Kilmarnock Infirmary last year, and there was only one death.

WRITERS to the *Times* are praising tobacco smoke as a disinfectant. It is even said to retard the spread of cancer.

THE picture of two dogs, called "Out-patients of King's College Hospital," has been purchased by Messrs. Pears.

THE Glasgow Charity Organisation Society has investigated 5,098 cases during the past year, and granted aid in 1,172 cases.

THE Wolverhampton Hospital Sunday Fund has been awarded as follows: General Hospital, £584; Eye Hospital, £52.

IT costs £60 a-year to keep a patient at the Leamington Incurable Hospital, and £70 a-year at a similar institution at Putney.

THE Zenana ladies of Lahore addressed their parting letter to Lady Dufferin, "Dear Mother of the Benighted Females of India."

THE Thomas Walker Hospital, Paramatta River, Australia, is to be built on the pavilion system from plans drawn by Mr. Kirkpatrick.

THE chapel of St. Barnabas Cottage Hospital, Saltash, has a dedication picture, depicting St. Barnabas, painted by Mr. E. Fellows Prynne.

THE decorations at Christmas in many of the Eye Hospitals were wasted so far as the patients were concerned, for few could see them.

W. A. WRIGHT, President of the Lincoln Co-operative Educational Committee, has presented thirty books to the Lincoln County Hospital.

THERE are 40 scarlet fever patients in the Sheffield Fever Hospital, and one typhoid patient. Last Christmas there were 150 small-pox patients.

AMONGST the most prominent of the large buildings being erected in Shaftesbury Avenue is the New French Hospital, which is to contain 52 beds.

AT the Royal Free Hospital, Gray's Inn Road, a performance of "Alice in Wonderland" was given on New Year's Eve by the lady medical students.

DUMBARTON is to have a Cottage Hospital. The plans have been chosen, and Messrs. Peter Denny and Walter Brock have promised subscriptions of £500 each.

MR. GEORGE GURNEY, honorary treasurer of the Princess Alice Hospital at Eastbourne, has been appealing to the public for a sum of £1,500 necessary for its enlargement.

AT Belfast Children's Hospital, not only did every little patient receive a present on Christmas Day, but the nurses and household staff also received gifts from the Ladies' Committee.

HOSPITAL Saturday Fund at Exeter has been divided as follows: General Hospital, £330; Eye Infirmary, £25; Dispensary, £55; Dental Hospital £10; Homœopathic Dispensary, £12.

SIXTY-FIVE cases, seven being scarlet fever, were treated in the Ayr Fever Hospital during last year, only one of which died—a malignant case, in which death occurred four days after admission.

THE Chicago Medical Society is convinced that Dr. Samuel Guthrie, of America, was the discoverer of chloroform. Does any other responsible body share the conviction of the Chicago Medical Society?

MRS. GEORGE FISHER PRIDEAUX has given the committee of the Bristol Dispensary a donation of £32 2s. 6d. for the purpose of placing in the committee-room a window in memory of her late husband.

DR. SIMPSON, who for twenty-two years has been one of the honorary physicians to the Manchester Royal Infirmary, having resigned his post, has been appointed honorary consulting physician to the institution.

MRS. T. BLACK contributes to the *Queen* an article, entitled "How I Began my Cottage Hospital." This hospital has now been in existence for sixteen years, and is devoted to the treatment of ulcerated legs and eczema.

WHAT is "oat flight?" We don't know; but they want it as well as old linen at the Essex and Colchester General Hospital, and we hope that those who are better acquainted with the substance than we, will not fail to send some.

MR. WILLIAM RATHBONE, chairman of the Liverpool Training School and Home for Nurses, writes to the *Liverpool Courier*, asking for £200 to £300 more in annual subscriptions to support the institution, and found a pension fund for the nurses.

MR. R. G. BRIGGS writes to the *Sunderland Herald* to express his obligation to Dr. Bruce Low and Nurse Walker, of the Sunderland and North Durham Eye Infirmary, for the skill and unvarying kindness they showed to his son when a patient in that institution.

AT a meeting of the Society for the Study of Inebriety, Dr. James Stewart, of Bristol, read a paper in which he said that it was unwise to hold out any hope of permanent cure of the alcohol habit, unless the patient would voluntarily submit to treatment for a year.

IT has been customary for twenty-one years to have the annual meeting of the Glasgow Royal Infirmary on New Year's Day. The custom was inaugurated by Sir James Lumsden when he was Lord Provost of the City, and is loyally carried on by Sir James King, who at present occupies that position.

### AMUSEMENTS AND RELAXATION.

### NEW PUZZLE PRIZE SCHEME.

SECOND QUARTERLY COMPETITION COMMENCED JANUARY 5TH, ENDS MARCH 30TH, 1889.

1. Prizes will be awarded for the best Original Puzzles in Prose or Verse relating to any of the Social, Political, or Philanthropical topics of the day. Competitors are requested to *strictly* observe this rule, or their contributions will be discredited.
2. Puzzles may take any form which the ingenuity of the competitors suggest. Answers must accompany contributions, and the source of obscure or ambiguous terms be given.
3. No competitor will be allowed to take more than one prize during the Quarter.
4. Eight Prizes of Standard Books will be given, the choice of the book to be left to the winner. First, Second, Third, Fourth, and Fifth Prizes for best Original Puzzles, £1 1s., 15s., 10s. 6d., 7s. 6d., and 5s. each.
5. First, Second, and Third Prize for Solutions of 15s., 10s. 6d., and 5s. each.

N.B.—All contributions must reach the office, 38, Old Jewry, E.C., not later than the *First Post* on *Thursday* in each week.

### PUZZLES FOR SOLUTION.

**Second Week.**

No. 4. DOUBLE ACROSTIC.

Whene'er my primals time draws near,
My finals also will appear;
The joyful children shout with glee
At all the fun produced by me.

1. Carpenters have need of me.
2. My music will your hearts inflame.
3. Barren or fruitful this may be.
4. Of failure oft' I bear the blame.
5. Soldiers of old bore me with pain.
6. This ends, and then begins again.
7. A native of a distant isle.
8. A salt that moisture will restrain
9. A grudge best treated with a smile. PATIENCE.

No. 5. PUZZLE ALPHABET.—The A B C of the . . . . . . . . . . . . . .

| | |
|---|---|
| A—— | N—— noted it. |
| B—— broached it. | O—— ordered it. |
| C—— called for it. | P—— published it. |
| D—— dictated it. | Q—— quickened it. |
| E—— examined it. | R—— required it. |
| F—— fled from it. | S—— subscribed to it. |
| G—— gloried in it. | T—— taught it. |
| H—— hailed it. | U—— undertook it. |
| I—— inquired for it. | V—— valued it. |
| J—— joyed in it. | W—— welcomed it. |
| K—— kindled it. | X—— xemplified it. |
| L—— listened to it. | Y—— yearned for it. |
| M—— magnified it. | Z—— zested it. TINIE. |

**Answers to Eleventh Week Puzzles.**

No. 29. DOUBLE ACROSTIC.

C lothin G
H orro R
R emors E
I nsuranc E
S ubjec T
T apet I
M ansio N
A wnin G
S teadines S

No. 30, ORIGINAL ANAGRAMS.

"The compliments of the season."
"The Hospital Annual."

**Marks for Solution of Puzzles.**

Total Marks.—Ruffles (1), 16; Daisy, 7; Lady Clara (1), 17; Reldas (1), 14; Mater, 9; Condorcet, 7; Tinie, 12; Gentian (2), 20; Marguerite, 5; Patience, 7; Gretta, 4; Amicus, 7; Morico, 3; Scrooge (1), 18; Smut, 2; Thanet (1), 19; Jenny Wren 3; Lauriston, 1.

*The Figures in parentheses are Marks for the week ending Dec. 27.*

**Results of First Quarterly Prize Competition for Original Puzzles.**

Total Marks for Quarter.—Lady Clara, 78; Tinie, 71; Patience, 63; Morico, 61; Gretta, 58; Nipper, 54; Bijou, 42; Thanet, 30; Daisy, 23; G. D., 15; Hercules, 11; Boadicea, 7; Salford, 6; Dandy Dick, 5; John Gilpin, 4; A. M. E., 3.

*First Prize* (£1 1s.)—Lady Clara (Mrs. F. W. Hume, Oaklands, Addiscombe).

*Second Prize* (15s.)—Tinie (Miss H. Todd, Newbury, Berks).

*Third Prize* (10s. 6d.)—Patience (Miss C. F. Calise, Newbury, Berks).

*Fourth Prize* (7s. 6d.)—Morico. (Miss Marguerite N. M. Didier d'Ambléon, Mustapha, Redhill).

*Fifth Prize* (5s.)—Gretta (Miss Margaretta Parker, Effingham Villa, Stoke Newington).

**Results of First Quarterly Competition for Solution of Puzzles.**

Total Marks for Quarter.—Gentian, 20; Thanet, 19; Scrooge, 18 Lady Clara, 17; Ruffles, 16; Reldas, 14; Tinie, 12; Mater, 9; Daisy, 7; Condorcet, 7; Patience, 7; Amicus, 7; Marguerite, 5; Gretta, 4; Morico, 3; Jenny Wren, 3; Smut, 2; Lauriston, 1.

*First Prize* (15s.)—Gentian (Miss Ethel Grundy, Pendleton, Manchester).

*Second Prize* (10s. 6d.)—Thanet (Miss W. Chexfield, R. S. B. Infirmary, Margate).

*Third Prize* (5s.)—Scrooge (Miss C. S. Mills, Pendleton).

N.B.—Prize Winners are requested to send in the names of the Books they wish for to the "Prize Editor" at once.

**Notice to Correspondents.**

We hope to welcome all our old friends and many new ones to the New Quarterly Competition for the Composition and Solution of Original Puzzles, which commenced January 5th. Those competitors who sent in puzzles last week will have them credited in our next issue for the first week of the above competition.

[For Conditions, see THE HOSPITAL for December 15th, 1888.]

